# Categories of Intimate Partner Violence and Abuse Among Young Women and Men: Latent Class Analysis of Psychological, Physical, and Sexual Victimization and Perpetration in a UK Birth Cohort

**DOI:** 10.1177/08862605221087708

**Published:** 2022-04-26

**Authors:** Annie Herbert, Abigail Fraser, Laura D. Howe, Eszter Szilassy, Maria Barnes, Gene Feder, Christine Barter, Jon Heron

**Affiliations:** 1 Department of Population Health Sciences, 1980 University of Bristol, Bristol, UK; 2 MRC Integrative Epidemiology Unit, 1980 University of Bristol, Bristol, UK; 3 Centre for Academic Primary Care, 1980 University of Bristol, Bristol, UK; 4 6723 University of Central Lancashire, Preston, UK

**Keywords:** youth violence, violence exposure, stalking, sexual assault, dating violence

## Abstract

**Background:**

In the UK, around one-third of young people are exposed to Intimate Partner Violence and Abuse (IPVA) by 21 years old. However, types of IPVA victimization in this population (psychological, physical, sexual), and their relationship with impact and perpetration are poorly understood.

**Methods:**

Participants in a UK birth cohort reported IPVA victimization and perpetration by age 21. We carried out a latent class analysis, where we categorized IPVA by types/frequency of victimization, and then assigned individuals to their most probable class. Within these classes, we then estimated rates of reported: 1) types of negative impacts (sad, upset/unhappy, anxious, depressed, affected work/studies, angry/annoyed, drank/took drugs more); 2) types/frequency of perpetration.

**Results:**

Among 2130 women and 1149 men, 32% and 24% reported IPVA victimization (of which 89% and 73% reported negative impact); 21% and 16% perpetration. Victimization responses were well represented by five classes, including three apparent in both sexes: *No-low victimization* (characterized by low probabilities of all types of victimization; average probabilities of women and men belonging to this class were 82% and 70%); *Mainly psychological* (15% and 12%); *Psychological and physical victimization* (4% and 7%), and two classes that were specific to women: *Psychological and sexual* (7%); *Multi-victimization* (frequent victimization for all three types; 4%). In women, all types of negative impact were most common in the *Psychological and sexual* and *Multi-victimization* classes; for men, the *Psychological and physical* class. In women, all types of perpetration were most common for the *Mainly psychological*, *Psychological and physical* and *Multi-victimization* classes; in men, the *Mainly psychological* and *Psychological and physical* classes.

**Discussion:**

In this study of young people, we found categories of co-occurrence of types and frequency of IPVA victimization associated with differential rates of negative impact and perpetrating IPVA. This is consistent with emerging evidence of IPVA differentiation and its variable impact in other populations.

## Background

Among young people in the UK, it is estimated that one-third to three-quarters are exposed to Interpersonal Violence and Abuse (IPVA) victimization by 21 years old, and one fifth perpetrate IPVA (Barter, 2009; Herbert et al., 2021; Young et al., 2018, 2019). Evidence mainly from north America suggests poor mental and physical outcomes in young people who experience IPVA (Goncy et al., 2017; Haynie et al., 2013; Sessarego et al., 2021), and so effective interventions for its prevention and related outcomes are needed (Barter & Stanley, 2016; Campbell et al., 2002).

Yet patterns of IPVA victimization in the UK context are poorly understood. Researchers have measured the prevalence of IPVA in the UK either as a binary concept, or of different types such as psychological or physical IPVA, alone (Barter, 2009; Herbert et al., 2021; Young et al., 2018, 2019), but we do not yet know the extent to which different types of IPVA co-occur for the same individual. Understanding co-occurrence and the potential differential impact of different combinations of abuse is crucial for developing interventions to support people experiencing IPVA and for its prevention. We searched for studies that reported on co-occurrence patterns of IPVA (search strategy provided in Supplementary Box S1), which identified a number of IPVA studies of young people, that were all from north America and all used Latent Class Analysis (LCA) (Brooks-Russell et al., 2013; French et al., 2014; Goncy et al., 2017; Haynie et al., 2013; Hebert et al., 2018; Lapierre et al., 2019; Martin-Storey & Fromme, 2016; Mumford et al., 2019; Sessarego et al., 2021; Swartout et al., 2012; Weir & Kaukinen, 2019). These studies identified victimization profiles, which differed between younger and older adolescents, estimated differential rates of negative emotional impact of victimization experiences and health outcomes between fitted classes, and found differences in relationships between victimization profiles and outcomes between the sexes. We identified no UK-based studies that have studied co-occurrence of IPVA types to this level. Findings from north American population studies, that have been principally carried out in student samples, and where the social and educational context greatly differs (e.g. State-specific vs. country-wide policies, different levels of school-based intervention for interpersonal violence and abuse) (Campbell, 2002; Fellmeth et al., 2013; Meiksin et al., 2019), as do modes of violence (e.g. the prevalence of violence through gun crime), cannot be assumed to be validly extrapolated to UK populations.

In addition, it has been argued that severity, frequency, and impact of IPVA are important aspects of IPVA to consider, particularly in exploring IPVA by sex (Bacchus et al., 2018; Hegarty et al., 2012; Hester et al., 2010; Walby & Allen, 2004; Walby & Towers, 2018). For example, prevalence of psychological IPVA may not be ‘gendered’, but the potential health damage of continuous coercive control is (Finkelhor et al., 2007; Herbert et al., 2021; Walby & Towers, 2018). A more differentiated categorization of IPVA victimization, considering types, severity, frequency and impact, is needed to evaluate its impact on individuals and families.

There is a need to understand how different types of IPVA victimization present in young people specifically in the UK, ideally considering frequency and severity, and whether these patterns vary in terms by age, sex and other outcomes.

Against this background, we investigated patterns of IPVA victimization experiences among young people aged up to 21 in a large UK population-based birth cohort according to types of abuse (psychological, physical, sexual), severity (e.g. coercive vs. forced sexual) and frequency. We then explored relationships between these patterns and different types of self-reported negative impact and IPVA perpetration.

## Methods

### Participants and Data Collection

We analysed data on 3279 young people who were part of the ALSPAC (Avon Longitudinal Study of Parents and Children, formerly ‘Children of the 90s’) birth cohort study, and who had answered questions relating to IPVA (delivered in online and paper form) at 21 years old. Demographic, behavioural, and health characteristics of this sample are described in Herbert et al., Extended Data, Table C (Herbert et al., 2021). Briefly, two-thirds were female, with a median age of 21 years (interquartile range: 21–22); women and men were more likely to be of low socioeconomic deprivation levels than high (e.g. 22% of women were in the quintile of Index of Multiple Deprivation most indicative of affluence, whereas 8% were in the quintile most indicative of poverty, noting high amounts of missing data on this variable); the large majority of both women and men were white (both 95%), self-defined as heterosexual (at least 68% and 60%), with 92% indicating that they had been in a relationship by age 21 (Herbert et al., 2021).

ALSPAC recruited pregnant women resident in Avon, UK, with expected dates of delivery 1st April 1991 to 31st December 1992, as well as their partners and offspring (our analyses focus on the offspring at age 21). The initial number of pregnancies enrolled was 14,541, resulting in 13,988 children who were alive at 1 year of age. When the oldest children were approximately 7 years of age, an attempt was made to bolster the initial sample with eligible cases who had failed to join the study originally – resulting in 15,454 total enrolled pregnancies, of which 14,901 foetuses were alive at 1 year of age. More information on ALSPAC data is available in published cohort profiles (Boyd et al., 2013; Fraser et al., 2013; Northstone et al., 2019), and the study website, which contains details of all the data available through a fully searchable data dictionary and variable search tool (http://www.bristol.ac.uk/alspac/researchers/our-data/). Study data were collected and managed using REDCap electronic data capture tools hosted at University of Bristol (Harris et al., 2019). Ethical approval for the study was obtained from the ALSPAC Ethics and Law Committee and the Local Research Ethics Committees. Full details of the ALSPAC consent procedures are available on the study website (http://www.bristol.ac.uk/alspac/researchers/research-ethics/).

The analyses of the current study focus on participants answering the questionnaire wave at age 21. All eligible participants who could be contacted (*n* = 9359) were provided details of an online questionnaire in mid-December 2013, and then sent a series of up to four reminders at 3-week intervals, some of these reminders containing a paper version of the same questionnaire. 3,463 (37%) of those contacted responded to the questionnaire; 3,279 (35%) completed the IPVA section of the questionnaire.

The IPVA section of the questionnaire, the relevant parts of which are provided in Supplementary Box S2, was developed by IPVA researchers, based on previous UK and European questionnaires and the PROVIDE questionnaire (Barter, 2009b; Hester et al., 2015), and Home Office Definition of Domestic Violence and Abuse. The phrasing of questions and impact response options were co-designed with a Young Persons’ Advisory Group. The IPVA measure showed ‘high internal consistency’ (alpha=0.95), and strong evidence for uni-dimensionality in a study validating its psychometric properties (Yakubovich et al., 2019). The questions on IPVA were approved by the ALSPAC Ethics and Law Committee (ref: E201210).

### Definitions of Different Interpersonal Violence and Abuse Types

For either victimization or perpetration, psychological abuse was defined as ‘non-physical behaviour aimed at intentionally harming or controlling another person emotionally’ (Barter, 2009b), including coercive/controlling behaviour (‘Coercive psychological’) (UK Home Office, 2012). Explicit psychological abuse was considered verbal aggressions as opposed to coercive psychological abuse, which was considered potentially more subtle coercive/controlling behaviour without physical force, such as telling a partner where they were allowed to go (Table 1). Physical abuse was defined as any act where an individual intentionally attempts to harm another through physical means. Sexual abuse was defined as any sexual contact or behaviour occurring without explicit consent of the victim. Coercive sexual abuse was considered to capture situations where a partner was verbally pressured into sexual acts without physical force, as opposed to forced sexual abuse (where the partner was at some point physically forced).

**Table 1.** Intimate Partner Violence and Abuse (IPVA) question wording, labelling employed in the current study, and their associated frequencies.

Victimization		Women, *n* (% of 2130)		Men, *n* (% of 1149)	
Question Wording	Type Label in Current Study	Once	A Few times+	Once	A Few times+
‘Told you who you could see and where you could go and/or regularly checked what you were doing and where you were (by phone or text)?’	Coercive psychological	82 (4)	443 (21)	42 (4)	156 (14)
‘Made fun of you, called your hurtful names, shouted at you?’	Explicit psychological	130 (6)	485 (23)	40 (3)	175 (15)
‘Used physical force such as pushing, slapping, hitting or holding you down?’	Physical 1	147 (7)	229 (11)	46 (4)	64 (6)
‘Used more severe physical force such as punching, strangling, beating you up, hitting you with an object?’	Physical 2	59 (3)	86 (4)	22 (2)	10 (0.9)
‘Pressured you into kissing/touching/something else?’	Coercive sexual 1	86 (4)	162 (8)	10 (0.9)	16 (1)
‘Pressured you into having sexual intercourse?’	Coercive sexual 2	157 (7)	166 (8)	24 (2)	19 (2)
‘Physically forced you into kissing/touching/something else?’	Forced sexual 1	63 (3)	65 (3)	5 (0.4)	<5 (<0.4)
‘Physically forced you into having sexual intercourse?’	Forced sexual 2	75 (4)	40 (2)	5 (0.4)	<5 (<0.4)
**Perpetration**					
**Question wording**	**Type label in current study**				
‘Told them who they could see and where they could go and/or regularly checked what they were doing and where they were (by phone or text)?’	Coercive psychological	82 (4)	193 (9)	36 (3)	73 (6)
‘Made fun of them, called them hurtful names, shouted at them?’	Explicit psychological	92 (4)	278 (13)	44 (4)	120 (10)
‘Hit, slapped, kicked or otherwise physically hurt them?’	Physical	106 (5)	94 (4)	20 (2)	8 (0.7)
‘Pressured or forced them into kissing, touching, sexual intercourse or any other sexual activity when they did not want to?’	Sexual	<5(<0.2)	<5 (<0.2)	11 (1)	12 (1)

### Interpersonal Violence and Abuse Victimization Measures

Within the IPVA section of the questionnaire, participants were asked ‘How often altogether have any of your partners ever done any of the following to you and how old were you?’, with ‘by “partner”, we mean anyone you have ever been out with or had a relationship with, long-term or short-term (including “one-night stands”)’. The eight different examples of victimization that followed are presented in Table 1, along with short-hand labels that we employ in the current study.

Where we employed the labels *Physical 1* and *Physical 2*, *Coercive sexual 1* and *Coercive sexual 2*, and *Forced sexual 1* and *Forced sexual 2*, we considered them to represent different severities (2 being higher than 1) of physical, coercive sexual and forced sexual IPVA, respectively. We did not consider *Coercive psychological* or *Explicit psychological* to have a particular order in terms of severity between each other. *Physical 2* captures more severe acts of physical abuse such as hitting with an object, (compared to e.g. slapping, in Physical 1). *Coercive/forced sexual 2* capture acts of sexual abuse related to intercourse (compared to e.g. kissing or touching, in *Coercive/forced sexual 1*). We made no assumptions about ordering of severity when modelling the above eight variables in Latent Class Analysis (described later under ‘Statistical Analysis’), the purpose of the labelling is to aid with interpretation, only.

The options for frequency of the above eight events were: ‘never’, ‘once’, ‘a few times’ and ‘often’. We treated these variables as ordinal categorical responses, where ‘a few times’ and ‘often’ were combined into one category (given small numbers of endorsements to ‘often’).

For men, numbers of endorsements to the two questions relating to *Coercive sexual 2* and *Forced sexual 2* (i.e. relating to intercourse) were considered too small for these data to be included in analyses (19 men reported *Coercive Sexual 2* either ‘a few times’ or ‘often’, 6 for *Forced Sexual 2*). Therefore, although all eight victimization questions were included in analyses for women, these two sexual victimization questions were not considered any further in analyses for men (leaving the six remaining victimization questions).

### Interpersonal Violence and Abuse Perpetration Measures

Participants were then asked: ‘How often altogether have you done any of the following to any of your partners, and how old were you?’. The four different examples of perpetration (similarly worded to the examples of victimization, but due to questionnaire space, slightly more condensed) are presented in Table 1. It should be noted that questions about perpetration did not distinguish either whether the perpetration took place in the same intimate relationship as any victimization events, or whether it took place before, during, and/or after victimization events.

### Negative impact of Interpersonal Violence and Abuse

Following the eight victimization questions and four perpetration questions, participants were asked: ‘How did you feel after they did these things to you?’ with the following examples of negative impact given: ‘upset/unhappy’; ‘affected my work/studies’; ‘made me feel sad’; ‘anxious’; ‘made me drink more alcohol/take more drugs’; ‘angry/annoyed’; ‘depressed’. Each of these impact responses were binary yes/no variables. Participants could endorse multiple items, and two or more endorsements do not necessarily relate to the same victimization/perpetration event.

### Statistical Analyses

We carried out all analyses separately for women and men. A large part of the literature has focussed solely on violence against women and so sex-stratified analyses would allow comparison with existing literature (Campbell, 2002; Ellsberg et al., 2008; Garcia-Moreno et al., 2013; Vezina & Hebert, 2007; Yakubovich et al., 2018). Further, small numbers led us to only be able to analyse six of the eight victimization questions for men.

We carried out a bias-adjusted three-step latent class analysis (LCA) (Heron et al., 2015; Vermunt, 2010). First, we carried out an LCA on the eight victimization questions (six for men; as ordinal variables ‘Never’, ‘Once’, ‘A few times+’). An LCA involves fitting the data to a pre-assigned number of classes using a multivariate mixture model. Within each class there is an estimated probability per response variable (e.g. if the variables included were binary: the first class could represent a high probability of *Explicit psychological* victimization, low probability of *Physical 1*, etc., with the second class being characterized by a different set of probabilities for each of the victimization outcomes). Each individual in the analysis then has a posterior probability of belonging to each class, based on their observed responses. Then, we decided on the final class solution based on different indicators of goodness-of-fit, face validity, a possible sex-invariant solution, and utility (classes that would not be too small). We checked the entropy of the class solution to determine how reliably individuals could be assigned to the different classes in the solution. Further details of this process are provided in Supplementary Box S3.

In the second step, we assigned each of the study participants to one of the derived classes based on their modal posterior probability of membership.

Finally, in the third step, to explore how patterns of impact and perpetration varied between classes, we fitted a set of multivariate logistic regression models for each sex in turn. For these models, the dependent variables consisted either of the seven binary measures of impact, or four of perpetration, with class assignment included as an independent variable and correction (bias-adjustment) for misclassification error in the modal assignment (Heron et al., 2015; Vermunt, 2010). The study-specific three-step process is described in more detail in Supplementary Box S3. We prepared all data in Stata version 15.1, ran LCAs in Mplus version 8.4, and derived proportions of impact and perpetration types and created plots using R version 3.5.1. The Mplus and R scripts used for analyses are available at: https://github.com/pachucasunrise/IPVA_categories.

## Results

Of 2130 women and 1149 men who responded to questionnaires at age 21, 880 (41%) and 330 (29%) reported IPVA victimization, respectively. The most common victimization type was psychological (35% and 26%), followed by physical (18% and 10%), then sexual (18% and 5%) (Herbert et al., 2021). Among those reporting IPVA victimization, 89% of women and 72% of men and reported any negative impact. Of the 2130 women and 1149 men, 25% and 20% reported perpetration, respectively, the most common types again being psychological, followed by physical, then sexual.

### Interpersonal Violence and Abuse victimization profiles

Supplementary Box S3 and Supplemental Table S1 provide detail on decisions made and model diagnostics between different class solutions. Briefly, indicators were inconsistent in that they pointed to the optimal solution being five or six classes in women (based on Bayes Information Criterion [BIC] and Bootstrap Likelihood Ratio [BLRT], and Vuong-Lo-Mendell-Rubin [VLMR], respectively) and two or three classes in men (based on BIC, and BLR and VLMR tests, respectively). Based on choosing the highest number of classes per sex to avoid missing important variation, and on face validity and avoiding classes of small numbers, we chose to present the five and three-class solutions for women and men, respectively.

**Figure 1.**
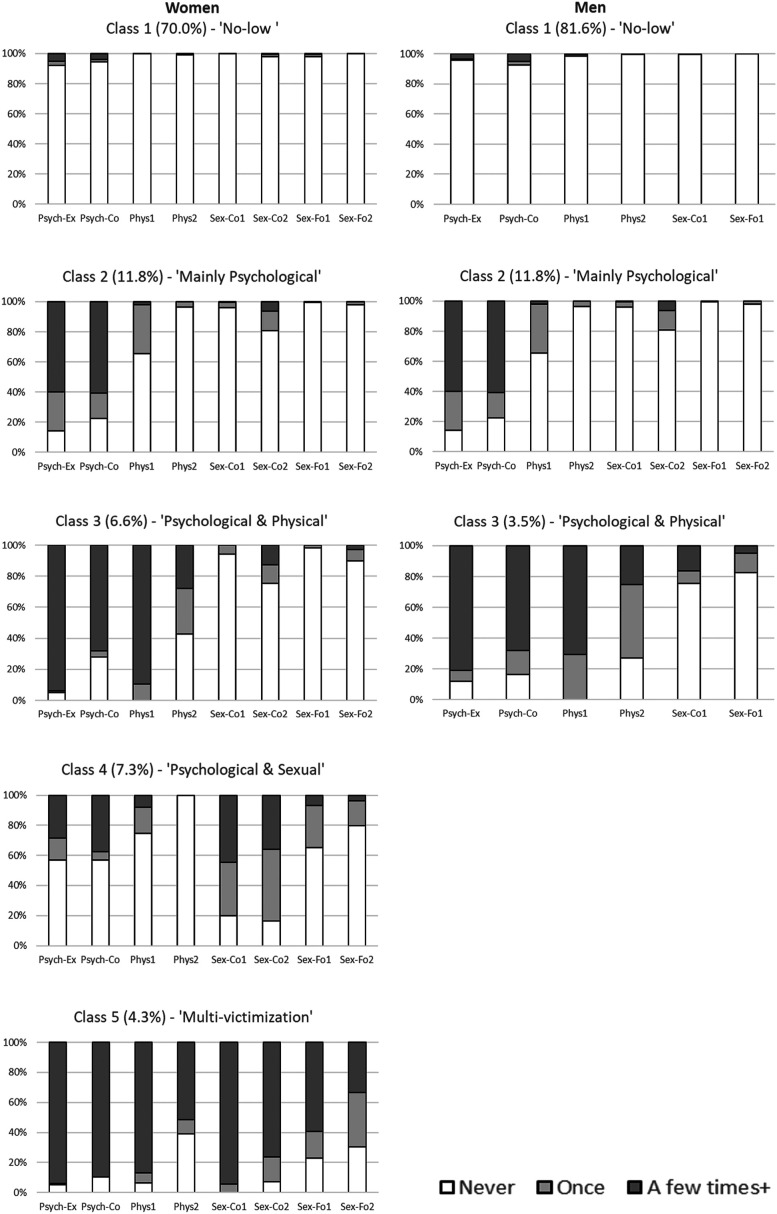
Five-class solution (women) and three-class solution (men) of victimization responses Psych-Co = *Coercive psychological*: told where you could go, who you could see, etc.; Psych-Ex = *Explicit psychological*: shouted at you, etc.; phys 1 = that is, pushing, slapping, etc.; Phys2: *Physical 2*, that is, punching, strangling, etc.; Sex-Co1: *Coercive sexual 1* such as pressured into kissing, touching, something else (not intercourse); Sex-Fo1: *Forced sexual 1* such as forced into kissing, touching, something else (not intercourse); Sex-Co2: *Coercive sexual 2* (intercourse); Sex-Fo2: *Forced sexual* (intercourse). Probabilities in parentheses of each plot represent the average probability (across all posterior probabilities in the sample) of belonging to that class.

The classes are presented in Figure 1. Classes 1–3 in the five-class solution in women were similar in terms of patterns of victimization types and frequency to Classes 1–3 of the three-class solution in men. However, note that these classes – in terms of exact probabilities of each of the types of victimization and their frequencies – though similar are not necessarily the same between sexes, as they were derived from separate models.

The classes were as follows:

Class 1: *Low-no victimization* (average posterior probabilities of belonging to this class were high: 70% for women and 82% for men). This class was characterized by small chances (0–5%) of (coercive or explicit) psychological, physical or sexual victimization.

Class 2: *Mainly psychological victimization* (average probabilities of belonging to this class, women: 12%, men: 15%). This class was characterized by frequent psychological victimization. Within this class there was an over 50% chance of frequent (i.e. at least ‘a few times’) explicit or coercive psychological victimization. At least 30% chance of experiencing physical victimization of the type *Physical 1* (i.e. ‘such as pushing, slapping, hitting or holding you down’).

Class 3: *Psychological and physical victimization* (women: 7%, men: 4%). Characterized by frequent psychological and physical victimization. Within this class there was an over 60% chance of frequent (i.e. at least ‘a few times’) explicit or coercive psychological victimization and of frequent physical victimization of the type *Physical 1* (i.e. ‘such as pushing, slapping, hitting or holding you down’). Over 50% chance of reporting at least ‘Once’ for *Physical 2* (i.e. ‘such as punching, strangling, beating you up, hitting you with an object’), which was more likely to occur ‘Once’ compared to ‘A few times+’.

Classes 4 and 5 related to women only:

Class 4: *Psychological and sexual victimization* (7%). Characterized by coercive sexual victimization, with a 36–48% chance of reporting it as being frequent (‘A few times+’; depending on whether type 1 or 2), and 35–48% of reporting it as having happened ‘Once’. This class was associated with over 40% chance of reporting either explicit or coercive psychological victimization, most of this being ‘A few times+’.

Class 5: *Multi-victimization* (4%). This class was defined by very high chances (>80%) of being explicitly or coercively psychologically victimized, physically victimized of the type *Physical 1* (i.e. ‘such as pushing, slapping, hitting or holding you down’), or coercively sexually victimized. There were also high chances (>50%) of being physically victimized of the type *Physical* 2 (i.e. ‘such as punching, strangling, beating you up, hitting you with an object’) or forcefully sexually victimized. In this class probabilities of being victimized frequently (‘A few times+’) were always much higher than ‘Once’.

Entropy was relatively high: 0.90 for the five-class solution in women and 0.86 for the three-class solution in men.

### Interpersonal Violence and Abuse Victimization Impact Outcomes

**Figure 2.**
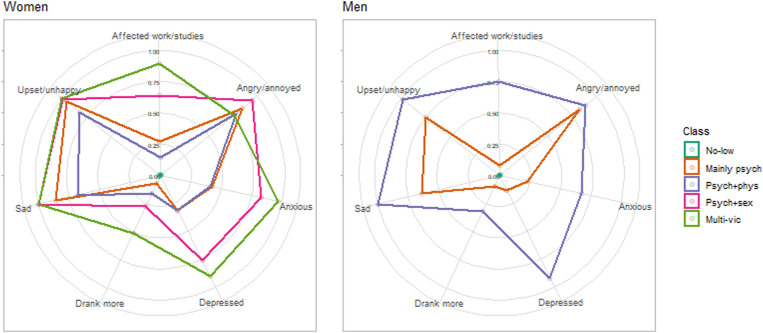
Rates of reported different types of negative impact by victimization class and sex. p-value for Wald test < 0.0001.

After assigning individuals to classes based on their modal posterior probability, the rates of different impact types by the five classes of women and three classes of men were as shown in Figure 2 (exact values for rates and standard errors are presented in Supplemental Table S2). Between the five classes in women, there was a general pattern of rates of negative impact being lowest in the *No-low victimization* class (class 1), increasing in the *Mainly psychological victimization* class (class 2), then *Psychological and physical victimization* class (class 3), *Psychological and sexual victimization* class (class 4), and finally the *Multi-victimization* class (class 5) (noting the increased surface area covered by each class in Figure 2). In men, rates of each type of negative impact were lowest in the *No-low victimization* class (class 1), increased in the *Mainly psychological victimization* class (class 2), followed a further increase in the *Psychological and physical victimization* class (class 3).

Within each class and regardless of sex, the rates of negative impact tended to be highest for ‘Angry/annoyed’, ‘Upset/unhappy’ and ‘Sad’, and the lowest for ‘Made me drink more alcohol/take more drugs’.

### Interpersonal Violence and Abuse Perpetration Outcomes

**Figure 3.**
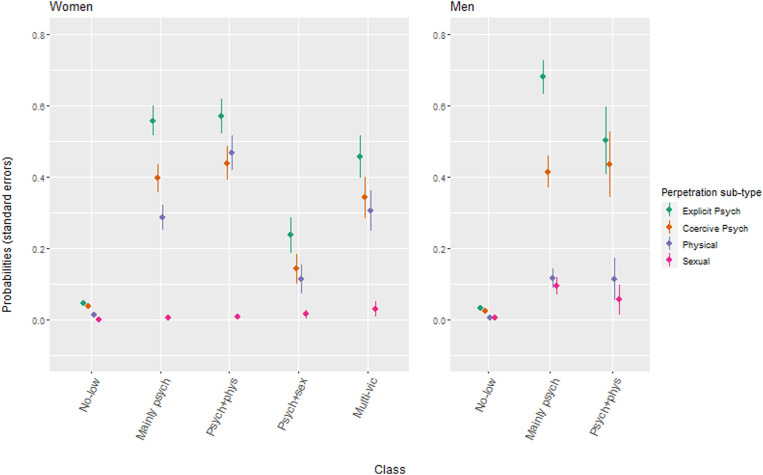
Rates of reported types of perpetration*, by victimization classes and sex%*A response of at least ‘Once’. p-value for Wald test < 0.0001.

The rates of different perpetration types by victimization classes are shown in Figure 3 (exact values for rates and standard errors are presented in Supplemental Table S3).

In women, rates of explicit psychological, coercive psychological and physical perpetration, were generally highest in the *Mainly psychological victimization* class (class 2), *Psychological and physical victimization* class (class 3) and *Multi-victimization* class (class 5), and lowest in the *No-low victimization* class (class 1). Rates of sexual perpetration were extremely low for all classes in women (<5%).

In men, rates of different perpetration types were higher in the *Mainly psychological victimization* class (class 2) and *Psychological and physical victimization* class (class 3) when compared with the *No-low victimization* class (class 1). There were no notable differences in rates between the *Mainly psychological victimization* class (class 2) and *Psychological and physical victimization* class (class 3), except that the risk of explicit psychological perpetration was higher in the *Mainly psychological victimization* class (class 2).

Within each class and regardless of sex, the rates of different perpetration types were highest for explicit psychological perpetration and the lowest for sexual perpetration.

## Discussion

This is one of few contemporary studies, and the first UK one, to articulate categories of co-occurrence of IPVA victimization types (psychological, physical and sexual) and frequency among young people, and to address co-occurrence of victimization with either negative impact and/or perpetration.

In this study we used a bias-adjusted three-step latent class analysis (LCA) to identify classes of young people according to exposure to different types and frequency of psychological, physical and sexual victimization by age 21, and estimate the association between these classes with negative impact and perpetration. We identified five classes: three classes that were similar for women and men were (in order of most likely classes): *No-low*, *Mainly psychological*, and *Psychological and physical* victimization; two classes specific to women were: *Psychological and sexual* and *Multi-victimization*. For both sexes, all seven types of reported negative impact were increasingly likely in this order (from *No-low* to *Multi-victimization*). For women, all types of perpetration were most common for the *Mainly psychological*, *Psychological and physical* and *Multi-*victimization classes; for men, they were most common in the *Mainly psychological* and *Psychological and physical* victimization classes.

We can only broadly compare our findings to previous (north American) LCA studies, given the different victimization items included, how they were captured (different questionnaire scales, and asking about different time frames of past 2 months to past year, compared to ‘ever’ in our study), and given that in previous studies perpetration was often included in models simultaneously to victimization (Goncy et al., 2017; Haynie et al., 2013; Hebert et al., 2018; Sessarego et al., 2021). Nevertheless, there were high-level similarities. We reported a no-low and a multi-victimization class, with similar rates of probable membership (77% and 4%, respectively) to two studies in adolescents with an average age of 15 (Hebert et al., 2018; Sessarego et al., 2021), except that probabilities associated with the multi-victimization class in our study were noticeably lower (4% for girls and 4% for boys vs. 12% and 7% previously) (Hebert et al., 2018). We also, like previous studies, found varied rates of negative impact of victimization experiences, including some not explored before (e.g. effect on work/studies). Unlike previous studies, we found that *psychological and sexual victimization* and *multi-victimization* classes were specific to females. It is possible that this is specifically the case in emerging adulthood (the majority of IPVA events in this sample took place at age 18-21) (Herbert et al., 2021), in a UK population, or when frequency is considered. Indeed, previous work from UK surveys found that women experience greater frequency of violence and abuse than men, and thus not taking frequency into account under-estimates the differences in experiences between the sexes (Walby & Towers, 2018). Our findings of increased negative impact for the *Psychological and sexual victimization* class compared to *No-low* in young people aged up to 21, and even further increased risk for the *Multi-victimization* class, correspond with what has been found previously in high-school adolescents (Goncy et al., 2017).

### Implications

There is increasing interest from researchers, service providers and policymakers, regarding IPVA among young people. (Herbert et al., 2021). Findings will be relevant for understanding victimization patterns in UK populations. Though they cannot be generalised to other populations in the Global North (e.g. north America, the rest of Europe), they can provide insights, for example helping to interpret future evidence from other high-middle income countries on interventions or outcomes.

The reported five distinct classes of young people, who differed in their likelihood of exposure to different types of psychological, physical, and sexual IPVA victimization, can inform the design of services dealing with young people exposed to IPVA and within future research. Psychological victimization was a feature for all classes other than *No-low victimization* (i.e. classes 2–5), and so identification of physical or sexual victimization is likely to signal presence of psychological victimization, too. This is consistent with other research on intimate partner violence among adults (Ansara & Hindin, 2010; Bailey et al., 2018; Potter et al., 2020), and indicates that although young relationships takes place in a different context to older ones, for example, the individual is less likely to cohabit with their partner (Theobald et al., 2016), psychological victimization can still be as pervasive. Despite difficulties with IPV measurement (Walby et al., 2016), current statistics on different patterns of IPVA types, severity, and frequency, can provide a greater understanding of the extent of repeat victimization, and confirm to those victimized how common their own experiences are. Further, we show a ‘dose-response’, in both sexes but particularly among women, between classes that represent different numbers of types/frequency/severity of IPVA and increased negative impact. It is feasible that a higher intensity of IPVA would result in higher likelihood of negative impact. It is also possible that an individual may more easily be able to recognize and attribute a link between a more intense IPVA exposure and their other negative experiences. This dose–response relationship has implications for those wanting to identify IPVA cases, as those victimized may find it easier to communicate the impact of the IPVA, rather than the IPVA itself.

Given the three classes that were similar between women and men, our findings support a hypothesis of gender symmetry in experiencing IPVA that involve frequent psychological and/or physical but not sexual aggression, but gender asymmetry in experiencing the most chronic IPVA multi-victimization or any sexual aggression (Ansara & Hindin, 2010). For young women there were two classes that were not apparent for young men: *Psychological and sexual* (class 4) and *Multi-victimization* (class 5), both characterized by likely sexual victimization; there was an average probability of 11% among the women in the study sample, of belonging to either of these classes.

We also provide an indication as to whether the category of IPVA types/frequency affects whether the person experiencing abuse perceives the IPVA as harmful or abusive. Each type of negative impact was least likely for the *No-low* victimization class and the most likely for those exposed frequently to the most types of victimization (women in the *Multi-victimization* class [class 5] and men in the *Psychological and physical* victimization class [class 3]) (Figure 2). That is, a large majority of those exposed to IPVA perceive the behaviour as harmful, and roughly in a ‘dose-response’ manner. This variation in impact also suggests how consequential it may be to distinguish types, frequency, and severity in future longitudinal cohorts measuring IPVA.

Our findings suggest a ‘gendered’ relationship for victimization and perpetration rates. For women, though rates were similar between the *Mainly psychological*, *Psychological and physical* and *Multi-*victimization classes, they were noticeably lower in the *Psychological and sexual* victimization class (class 4). In contrast, in men, rates of self-reported perpetration did not vary by patterns of self-reported victimization that were not *No-low*’. This indicates that the mechanisms behind perpetration are more likely to vary in women. Qualitative work, that is currently in progress (http://www.bristol.ac.uk/primaryhealthcare/researchthemes/yarah-study/), has explored the possible mechanisms behind such perpetration (e.g. self-defence).

### Strengths and Limitations

This study was carried out in a large contemporary cohort of young people within the UK. Young adults are a particularly understudied group in IPVA or ‘dating violence’ (Jennings et al., 2017); the victimization questions cover all ages up to 21 years, but previous work has shown that the majority of experiences will be at age 18–21 (Herbert et al., 2021). These data have allowed us to study the relationship between victimization patterns with negative impact and perpetration types, the latter two variables rarely being available within the same dataset (Barter, 2009a; Capaldi et al., 2012; Jennings et al., 2017; Stonard et al., 2014; Vagi et al., 2013).

We used LCA to determine likely categories of victimization types; unfortunately, indicators for goodness-of-fit were not consensual on the optimal number of victimization classes. However, five was the largest number of classes where classes were of an acceptable size; a smaller number would increase the chance of missing important variation in victimization responses. These five profiles were plausible given our expert knowledge of IPVA.

Previous work in this cohort indicates that the ALSPAC cohort over-represents relatively affluent, predominantly White UK populations (Boyd et al., 2013). However, prevalence of IPVA in this cohort is similar to the wider UK general population, and other high income countries (Herbert et al., 2021; Stonard et al., 2014; Young et al., 2018). While there may be limited generalizability of average probabilities of the reported five classes, the classes' properties themselves are unlikely to differ (Howe et al., 2013). 

The questions used to identify cases of IPVA did not distinguish between short- or long-term relationships (including whether a one-night stand). The questions were developed in this way during previous research, which included interviews, that found that young people were less likely to distinguish between and label these different types of relationships as such (Barter, 2009b; Stanley et al., 2018). This will mean that the same class, for example, *Psychological and sexual* (Class 4 in women) could comprise very different relationship contexts, noting that the individual questions do still distinguish repeat victimization and whether the psychological/sexual abuse was coercive or explicit/forced.

When interpreting results regarding victimization or perpetration probabilities within each of the five classes, assumptions cannot be made about the sex or gender identity of either the person victimized or of the person who perpetrated the IPVA for the event in question. We did not restrict any analyses by sexual orientation (at least 8% of men and 9% of women were identified as not being 100% heterosexual at age 15, but 25–30% of data were missing) (Herbert et al., 2021), as this would likely be a poor representation of whether the intimate experience(s) where violence and abuse occurred was a same-sex one, given that sexual orientation (which is subject to change) was asked about at one time-point (age 15), and IPVA events were asked about retrospectively at age 21. We also did not explore gender identity, as this had not been asked about by age 21. Exploring IPVA patterns by sexual orientation and gender identity in-depth would be an important separate enquiry, given that previous research has highlighted different IPVA patterns and perceptions of IPVA among these groups (Dickerson-Amaya & Coston, 2019; Yakubovich et al., 2021). In our qualitative work, we have captured the experiences of socio-economically deprived individuals and ethnic and sexual orientation minorities, which is being used to inform as to how different experiences of different types, severity and frequency of IPVA, and its impact might be for these groups, compared to those reported in the current study.

It must be stressed that the data used provide little information on the context in which the IPVA occurred in. For example, reported profiles use information on involvement in victimization and perpetration, but not the relationships that this occurs in. We cannot assume that different types/severity, or total frequencies within these types, are occurring within the same intimate relationship, especially as this indicates occurrence at any time up to the age of 21. For example, it is possible that the *Multi-victimization* class represents victimization by multiple perpetrators, or that the corresponding frequent types of perpetration are being inflicted on different intimate partners. Similarly, we cannot assume temporal ordering of victimization categories. For example, the *Psychological and physical victimization* class can just as much represent psychological victimization followed by physical victimization, as vice versa. Our current qualitative work aims to capture such dynamics within these classes, within specific relationships, as well as context beyond the relationship (e.g. involvement of family members). Such work can provide further insight about the differences between victimization profiles, and how these differences relate to impact and perpetration outcomes.

The negative impact questions represent participants’ *perceived* negative impact of IPVA. Perceived impact gives some indication as to whether the person being victimized perceived the behaviour as harmful or abusive, as this is not always the case (Hearn, 2013; Hester et al., 2015). Indeed, in other parts of the questionnaire over 20% of women and 10% of men reported neutral or positive impacts (e.g. ‘no effect/not bothered’, ‘thought it was funny’) (Herbert et al., 2021), and therefore though they experienced negative impacts of the abuse at the time, may not have attributed it to the events/behaviours they had been asked about. There is likely to be some error in capturing even perceived impact through these questions: if the events occurred at a young age (i.e. enough time had passed since they were asked the questions at age 21), answers to the questions may be subject to recall bias (more so than remembering whether any victimization or perpetration ever occurred).

Perpetration is probably under-reported, particularly among men, and likely driven by factors such as social desirability, shame, guilt or embarrassment (including through challenging masculinity) (Chan, 2011; Malhi et al., 2020; Myhill, 2015). This is likely to attenuate any association between IPVA profiles and perpetration type, and so the relationships described in this report (e.g. those in Figure 3), give a lower bound to the likely ‘true’ relationship. Future research that can combine different linked modes of reporting among young people, such as previous work that has combined survey and interview data from the same sample (Bacchus et al., 2018), can indicate to what extent this relationship can be explained by different patterns of under-reporting between victimization classes.

## Conclusion

In this study of young people, we identified five distinct profiles of young UK people according to their IPVA victimization patterns, including two specific to women. These profiles capture the co-occurrence of different types and frequency of IPVA and identify groups with differential rates of negative impact of IPVA and of perpetrating IPVA. This is consistent with emerging evidence of IPVA differentiation and its variable impact in other populations.

## Supplemental Material

sj-pdf-1-jiv-10.1177_08862605221087708 – Supplemental Material for Categories of Intimate Partner Violence and Abuse Among Young Women and Men: Latent Class Analysis of Psychological, Physical, and Sexual Victimization and Perpetration in a UK Birth Cohort Supplemental Material, sj-pdf-1-jiv-10.1177_08862605221087708 for Categories of Intimate Partner Violence and Abuse Among Young Women and Men: Latent Class Analysis of Psychological, Physical, and Sexual Victimization and Perpetration in a UK Birth Cohort by Annie Herbert, Abigail Fraser, Laura D. Howe, Eszter Szilassy, Maria Barnes, Gene Feder, Christine Barter and Jon Heron in Journal of Interpersonal Violence
