# Supplementary material for: Categories of Intimate Partner Violence and Abuse Among Young Women and Men: Latent Class Analysis of Psychological, Physical, and Sexual Victimization and Perpetration in a UK Birth Cohort
Source: J Interpers Violence. 2022 Apr 26;38(1-2):931–54. doi: 10.1177/08862605221087708 (PMC9727413; doi:10.1177/08862605221087708)
Supplement: sj-pdf-1-jiv-10.1177_08862605221087708 – Supplemental Material for Categories of Intimate Partner Violence and Abuse Among Young Women and Men: Latent Class Analysis of Psychological, Physical, and Sexual Victimization and Perpetration in a UK Birth Cohort [file sj-pdf-1-jiv-10.1177_08862605221087708.pdf]

Supplementary File

Box S1: Search strategy used for identifying studies of co-occurrence of different types of Intimate Partner Violence among young people

We searched PubMed for any publications up to 17th December 2020,
using the terms:

(‘profile’ OR ‘latent’ OR ‘class’ OR ‘mixture’ OR ‘cluster’)[Title]
AND
(‘violence’ OR ‘abuse’ OR ‘assault’ OR ‘victim’)[Title]

which returned 2,616 results.

After scanning titles and abstracts of these results, we identified 11 publications that reported on co-occurrence of different types of intimate partner violence and abuse among young people (aged up to and including 25), either exclusively, or at least as a separate age-group from a larger study sample (Brooks-Russell, Foshee, & Ennett, 2013; French, Bi, Latimore, Klemp, & Butler, 2014; Goncy et al., 2017; Haynie et al., 2013; Hebert, Moreau, Blais, Oussaid, & Lavoie, 2018; Lapierre, Paradis, Todorov, Blais, & Hebert, 2019; Martin-Storey & Fromme, 2016; Mumford, Liu, & Taylor, 2019; Sessarego et al., 2019; Swartout, Cook, & White, 2012; Weir & Kaukinen, 2019).

Box S2: Intimate Partner Violence and Abuse questionnaire

The full questionnaire (including sections not about IPVA) is available here: http://www.bristol.ac.uk/media-library/sites/alspac/documents/questionnaires/20131213%20YP%20Your%20Life%20Now%20Questionnaire.pdf

Section E: Intimate Partner Violence

The following section is about partner violence, sometimes called domestic abuse. We know this is a sensitive subject, but it is important to ask as it is not uncommon. Please remember that all answers are confidential. You do not have to answer any of these questions if you do not want to.

By 'partner', we mean anyone you have ever been out with or had a relationship with, long-term or short-term (including 'one night stands').

E1) **How often** altogether have any of **your partners** ever done any of the following to you and **how old were you**?

(cross one option on each line)

		1 Never	2 Once	3 A few times	4 Often
a)	Told you who you could see and where you could go and/or regularly checked what you were doing and where you were (by phone or text)?	1 ○	2 ○	3 ○	4 ○
	(cross all that apply) 1 ☐ Under 18	2 ☐ Over 18			
b)	Made fun of you, called your hurtful names, shouted at you?	1 ○	2 ○	3 ○	4 ○
	(cross all that apply) 1 ☐ Under 18	2 ☐ Over 18			
c)	Used physical force such as pushing, slapping, hitting or holding you down?	1 ○	2 ○	3 ○	4 ○
	(cross all that apply) 1 ☐ Under 18	2 ☐ Over 18			
d)	Used more severe physical force such as punching, strangling, beating you up, hitting you with an object?	1 ○	2 ○	3 ○	4 ○
	(cross all that apply) 1 ☐ Under 18	2 ☐ Over 18			
e)	Pressured you into kissing/touching/something else?	1 ○	2 ○	3 ○	4 ○
	(cross all that apply) 1 ☐ Under 18	2 ☐ Over 18			

36

(cross one option on each line)		[1] Never	[2] Once	[3] A few times	[4] Often
f)	Physically forced you into kissing/touching/something else?	[1] ○	[2] ○	[3] ○	[4] ○

(cross all that apply)

[1] ☐ Under 18	[2] ☐ Over 18

g)	Pressured you into having sexual intercourse?	[1] ○	[2] ○	[3] ○	[4] ○

(cross all that apply)

[1] ☐ Under 18	[2] ☐ Over 18

h)	Physically forced you into having sexual intercourse?	[1] ○	[2] ○	[3] ○	[4] ○

(cross all that apply)

[1] ☐ Under 18	[2] ☐ Over 18

i)	Did any of the above make you feel scared or frightened, or did any partner make you feel frightened in any other way?	[1] ○	[2] ○	[3] ○	[4] ○

(cross all that apply)

[1] ☐ Under 18	[2] ☐ Over 18

➡ **If you answered '<u>Never</u>' to ALL the above questions, please go to E3**

E2) How did you feel after they did these things to you?

(cross one option on each line)		[1] Yes	[2] No
a)	Upset/unhappy	[1] ○	[2] ○
b)	Affected my work/studies	[1] ○	[2] ○
c)	Made me feel sad	[1] ○	[2] ○
d)	No effect/not bothered	[1] ○	[2] ○
e)	Anxious	[1] ○	[2] ○
f)	Made me drink more alcohol/take more drugs	[1] ○	[2] ○
g)	Felt loved/protected/wanted	[1] ○	[2] ○
h)	Thought it was funny	[1] ○	[2] ○
i)	Angry/annoyed	[1] ○	[2] ○
j)	Depressed	[1] ○	[2] ○

Box S3. Study-specific details on the three-step Latent Class Analysis

We first ran a LCA on the eight victimization questions (six in the case of men). Variables were entered as ordinal variables (None, Once, and given numbers of responses in the more extreme categories, a combined response for A few times and Often). We ran analyses for possible solutions of two to six classes. We based our choice of optimal numbers of classes on several aspects:

- **Indicators of goodness-of-fit.** We used the Bayesian Information Criteria (BIC) and the p-values for the Bootstrap Likelihood Ratio Test (BLRT) and Vuong-Lo-Mendell Rubin (VLMR) test, to guide our decision about number of classes.(Morgan, 2015; Nylund, Asparoutiov, & Muthen, 2007) In the case of BIC, the lowest value indicated best model fit, i.e., best fitting number of classes. A small p-value [in our case, pre-determined threshold of 0.005] for the BLRT and LMR Test, respectively, of a model for C classes indicates a better fit than a model for C-1 classes. It should be noted that in previous methodological work comparing the BIC, and BLRT and LMR Tests, the BLRT performed better than both the BIC and LMR at correctly specifying numbers of classes for LCA of categorical variables, and so when these indicators disagreed between the 'true' number of classes, that indicated by the BLRT would take precedent.(Nylund et al., 2007) These model diagnostics are provided in **Supplementary Table S1**, which indicated very different sets of optimal classes. The BIC indicated that the best solutions would be two-class in men and five-class in women, the BLRT that this would be three-class in men and five-class in women, and the VLMR test the three-class in men and six-class in women. In this situation, we would decide to take the highest numbers of classes possible (i.e., three-class solution in men and six-class in women), as to not miss important variation in the independent variables. This is so long as it is not at the expense of face validity, a possible sex-invariant solution, or utility (each discussed below).
- **Face validity.** Co-authors on this paper (CB, ES, and GF) are experts in the field of interpersonal violence, and so given multiple indications in terms of optimal number of

classes (e.g. BIC indicates one number and BLRT another), they studied the classes to determine which made more sense in terms of context and previous research findings.

- **Sex invariance.** A-priori, where possible, we attempted to establish if there were any sex-invariant classes, as this allows for comparisons of the same set of classes between females and males, particularly for purposes of studying the relationship between profiles and other variables, such as impact and perpetration. Each model diagnostic indicated different numbers of classes between sexes (**Supplementary Table S1**), however the classes in the three-class solution in men appear to show similar patterns to three of the five-class and six-class solutions in women (**Figure 1**).
- **Utility.** We aimed to choose a solution where classes and their probabilities would be of an adequate size size to provide stability to estimates. For example, in the six-class solution in women, several probabilities associated with this class would represent less than 5 individuals.

Entropy represents how separate or distinct classes are in terms of their features (i.e., sets of probabilities of responses), i.e., how well the model can classify individuals given these classes. The 'best' model can still have a low entropy given a real overlap between the 'true' classes. Therefore, while we didn't base ideal number of classes on entropy, we report it for a better understanding of how well we could classify individuals based on their data. Generally, it is accepted that an entropy (which can range from 0 to 1) of at least 0.8 represents good separation. For the one-six classes solutions in men and women, entropy was at least 0.86 (ranging up to 0.91)

Table S1: Model diagnostics between different numbers of classes

	Women			Men		
**Number of classes**	**BIC**	**BLRT**	**VLMR**	**BIC**	**BLRT**	**VLMR**
1	16165.02	N/A	N/A	4243.19	N/A	N/A
2	13215.15	< 0.0001	<0.0001	**3738.39**	< 0.0001	<0.0001
3	12721.31	< 0.0001	< 0.0001	3769.78	**< 0.0001**	**0.0001**
4	12479.59	< 0.0001	**0.0001**	3840.16	0.378	0.2479
5	**12437.93**	< 0.0001	1.0000	3914.43	0.674	0.9313
6	12442.39	**< 0.0001**	0.3506	3989.77	0.386	0.8584

Values tabulated are comparing the k-class versus (k-1) class solution. Values in bold font signify that that value indicates best model fit, for that indicator.
BIC = Bayes Information Criterion; BLRT = Bootstrap Likelihood Ratio Test; VLMR= Vuong-Lo-Mendell Rubin Test; N/A = Not applicable

Table S2. Estimated rates of negative impact types, between classes

		Women		Men	
**Victimization class**	**Impact type**	**Rate**	**Standard Error**	**Rate**	**Standard Error**
1 (No-low)	Angry/annoyed	1.6%	0.004	1.3%	0.004
	Sad	0.2%	0.001	0.0%	0.000
	Upset/unhappy	0.7%	0.003	0.4%	0.003
	Depressed	0.0%	0.000	0.0%	0.000
	Affected work/studies	0.0%	0.000	0.0%	0.000
	Anxious	0.1%	0.001	0.0%	0.000
	Drank more	0.1%	0.002	0.1%	0.001
2 (Psych)	Angry/annoyed	84.5%	0.023	83.1%	0.036
	Sad	86.1%	0.059	63.7%	0.052
	Upset/unhappy	95.6%	0.042	75.2%	0.045
	Depressed	31.4%	0.074	13.7%	0.038
	Affected work/studies	27.1%	0.064	8.2%	0.036
	Anxious	42.6%	0.072	23.0%	0.042
	Drank more	6.8%	0.023	9.7%	0.025
3 (Psych & phys)	Angry/annoyed	79.0%	0.061	89.0%	0.053
	Sad	67.7%	0.168	100.0%	0.000
	Upset/unhappy	81.9%	0.127	98.1%	0.020
	Depressed	31.3%	0.178	91.7%	0.099
	Affected work/studies	14.6%	0.158	75.4%	0.086
	Anxious	40.8%	0.172	67.2%	0.087
	Drank more	15.6%	0.042	31.8%	0.081
4 (Sex & psych)	Angry/annoyed	94.9%	0.023		
	Sad	100.0%	0.000		
	Upset/unhappy	98.9%	0.011		
	Depressed	75.8%	0.073		
	Affected work/studies	64.2%	0.073		
	Anxious	83.2%	0.066		
	Drank more	27.6%	0.054		
5 (Multi-vic)	Angry/annoyed	76.1%	0.051		
	Sad	100.0%	0.000		
	Upset/unhappy	100.0%	0.000		
	Depressed	90.5%	0.050		
	Affected work/studies	89.5%	0.053		
	Anxious	96.7%	0.034		
	Drank more	51.0%	0.074		

Table S3. Estimated rates of perpetration type*, by frequency

		Women		Men	
**Victimization class**	**Perpetration outcome**	**Rate**	**Standard Error**	**Rate**	**Standard Error**
1 (No-low)	Explicit psychological	4.7%	0.006	3.3%	0.008
	Coercive psychological	3.9%	0.006	2.4%	0.006
	Physical	1.2%	0.004	0.5%	0.003
	Sexual**	0.0%	0.000	0.5%	0.003
2 (Psych)	Explicit psychological	55.8%	0.041	68.0%	0.048
	Coercive psychological	39.6%	0.038	41.4%	0.045
	Physical	28.7%	0.035	11.5%	0.027
	Sexual**	0.4%	0.005	9.5%	0.025
3 (Psych & phys)	Explicit psychological	57.1%	0.048	50.2%	0.095
	Coercive psychological	43.9%	0.047	43.6%	0.092
	Physical	46.8%	0.049	11.4%	0.059
	Sexual**	0.8%	0.008	5.6%	0.042
4 (Sex & psych)	Explicit psychological	23.7%	0.050		
	Coercive psychological	14.2%	0.043		
	Physical	11.3%	0.040		
	Sexual	1.5%	0.013		
5 (Multi-victimization)	Explicit psychological	45.8%	0.060		
	Coercive psychological	34.2%	0.057		
	Physical	30.5%	0.056		
	Sexual	3.0%	0.022		

*A response of at least ‘Once’.

**In the case of men, this represented pressured or forced into kissing, touching, something else, but not intercourse.

References

Morgan, G. B. (2015). Mixed Mode Latent Class Analysis: An Examination of Fit Index Performance for Classification. *Structural Equation Modeling-a Multidisciplinary Journal, 22*(1), 76-86. doi:10.1080/10705511.2014.935751

Nylund, K. L., Asparoutiov, T., & Muthen, B. O. (2007). Deciding on the number of classes in latent class analysis and growth mixture modeling: A Monte Carlo simulation study. *Structural Equation Modeling-a Multidisciplinary Journal, 14*(4), 535-569. doi:Doi 10.1080/10705510701575396
